# Dandelion Extracts Protect Human Skin Fibroblasts from UVB Damage and Cellular Senescence

**DOI:** 10.1155/2015/619560

**Published:** 2015-10-20

**Authors:** Yafan Yang, Shuangshuang Li

**Affiliations:** ^1^Sir Winston Churchill Collegiate and Vocational Institute, Thunder Bay, ON, Canada P7C 1V5; ^2^Cardiovascular and Metabolic Research Unit, Lakehead University, Thunder Bay, ON, Canada P7A 7T1

## Abstract

Ultraviolet (UV) irradiation causes damage in skin by generating excessive reactive oxygen species (ROS) and induction of matrix metalloproteinases (MMPs), leading to skin photoageing. Dandelion extracts have long been used for traditional Chinese medicine and native American medicine to treat cancers, hepatitis, and digestive diseases; however, less is known on the effects of dandelion extracts in skin photoageing. Here we found that dandelion leaf and flower extracts significantly protect UVB irradiation-inhibited cell viability when added before UVB irradiation or promptly after irradiation. Dandelion leaf and flower extracts inhibited UVB irradiation-stimulated MMP activity and ROS generation. Dandelion root extracts showed less action on protecting HDFs from UVB irradiation-induced MMP activity, ROS generation, and cell death. Furthermore, dandelion leaf and flower but not root extracts stimulated glutathione generation and glutathione reductase mRNA expression in the presence or absence of UVB irradiation. We also found that dandelion leaf and flower extracts help absorb UVB irradiation. In addition, dandelion extracts significantly protected HDFs from H_2_O_2_-induced cellular senescence. In conclusion, dandelion extracts especially leaf and flower extracts are potent protective agents against UVB damage and H_2_O_2_-induced cellular senescence in HDFs by suppressing ROS generation and MMP activities and helping UVB absorption.

## 1. Introduction

When skin is aged, skin is more transparent, loose, and fragile. One of the most damaging actions on skin is from solar radiation, especially from its ultraviolet (UV) component, leading to both clinical and histologic damage on human skin [1, 2]. UV irradiation causes distinct alterations of the connective tissues by generating excessive reactive oxygen species (ROS) and induction of matrix metalloproteinases (MMPs). MMPs-mediated degradation of the collagenous extracellular matrix (ECM) accounts for most of the connective tissue damage that occurs in photodamaged skin [3, 4]. Dermal fibroblasts are responsible for generating ECM and allowing the skin to recover from injury [5].

UV irradiation consists of three components, UVA, UVB, and UVC. Whereas UVA and UVB reach the earth in sufficient amounts to damage the skin, UVC is almost completely absorbed by the ozone layer [2, 6]. UVB is particularly damaging, as it penetrates the epidermis and the upper part of the dermis, where it damages fibroblast cells and leads to sunburn, photoageing, and skin cancer [7, 8]. In Canada, sunlight is strong enough to cause skin cancer and premature ageing of the skin. People often use sunscreen to protect the skin from UV damaging from the sun, and sunscreen absorbs UV rays and prevents them from penetrating the skin. However some sunscreen ingredients, including oxybenzone, benzophenone, and octocrylene, have been shown to be potentially skin carcinogenic or penetrate into our body and have other health risks [9]. It is necessary to find effective, safer, and environmentally friendly nature products for antiageing and UV protection.

*Taraxacum officinale*, commonly known as dandelion, is widely distributed in the warmer temperate zone of the Northern Hemisphere [10]. Dandelion extracts have been used for centuries for traditional Chinese medicine and native American medicine to treat cancers, hepatitis, and digestive diseases [11–14]. Dandelion extracts are shown to have anti-inflammatory, antioxidant, and anticarcinogenic activities; however, there are few scientific studies to investigate the effects of dandelion extracts in the treatment of skin diseases, especially photoageing [10, 15].

The purpose of this study was to determine whether dandelion leaf, flower, or root extracts can protect human dermal fibroblasts (HDFs) from UVB damage and cellular senescence and the underlying mechanisms. We found that dandelion leaf and flower extracts but not root extracts are potent protective agent against UVB damage and H_2_O_2_-induced cellular senescence in HDFs by suppressing ROS generation and MMP activities.

## 2. Materials and Methods

### 2.1. Preparation of Dandelion Extract

Dandelions were collected at flowering stage in June-July 2013 from the house yards in Thunder Bay (latitude 48°22′56′′N; longitude 89°14′46′′W), Ontario, Canada. Leaf, flower, and root were separated, cleaned, and air-dried before extraction through 4 hours of boiling in distilled water [13, 16]. The water extracts were filtered through grade 1 Whatman filter paper under vacuum, concentrated, and then dissolved in distilled water following filtration through a 0.2 *μ*m filter to avoid cell contamination, and 300 mg/mL stock aqueous extract was prepared and stored at 4°C for experimental use. The identity of plant was confirmed by Dr. Wei Cao (Lakehead University), and the herbarium specimens were deposited in Cardiovascular and Metabolic Research Unit, Lakehead University for future reference.

### 2.2. Cell Culture

HDFs were obtained from American Type Culture Collection (Manassas, VA) and cultured with Dulbecco's modified Eagle's medium supplemented with 10% fetal bovine serum, 100 U/mL penicillin, and 100 *μ*g/mL streptomycin and grown in a CO_2_ incubator at 37°C in 5% CO_2_ [17, 18]. The experiments were performed when the cells reached 70–80% confluence between passages 4 and 8. In all studies, cells were incubated in the serum-free medium for 12 h, and then 10% serum added together with different treatments. The media were changed every three days.

### 2.3. Cellular Viability Assays

Cell viabilities were measured based on conversion of yellow tetrazolium salt 3-(4,5-dimethylthiazol-2-yl)-2,5-diphenyltetrazolium bromide (MTT) to dark blue formazan by viable cells [19, 20]. Briefly, cells at equal number were plated onto each well of 96-well plates for 24 h. After treatment, 100 *μ*L medium containing 5 mg/mL MTT was added to each well, and the cells were then cultured at 37°C for 4 h. The medium was then discarded and the crystals were dissolved in dimethyl sulfoxide and absorbance of formazan products at 570 nm was measured in a Multiskan spectrum microplate spectrophotometer (Thermo Labsystems, Franklin, MA). The cells incubated with control medium were considered 100% viable.

### 2.4. UVB Irradiation

HDFs were rinsed with phosphate buffered saline (PBS) and irradiated using a UVB lamp (UVB-18, Claremont, CA) with a wavelength range of 280–315 nm [3, 21]. UVB was administered for 60 seconds (200 mJ/cm^2^) and the cells were cultured for another 24 hours following detection of cell viability. Dandelion extracts were added 30 minutes before or immediately after UVB radiation. The control cells were untreated with UVB irradiation.

### 2.5. Measurement of ROS and Glutathione (GSH) Contents

2′,7′-Dichlorodihydrofluorescein diacetate (H_2_DCFDA) (Invitrogen, Carlsbad, CA) was used to detect ROS [13, 22]. After treatments, HDFs cultured in 35 mm plates were washed with PBS once and then incubated with 1 *μ*g/mL H_2_DCFDA in medium for 30 minutes at 37°C. Then the cells were rinsed twice in PBS, and the fluorescence signals were detected using a fluorescent microscope. ImageJ software was used to quantify fluorescent intensity. Total GSH contents were measured using a commercial GSH assay kit (Cayman Chemical, Ann Arbor, MI), as previously described.

### 2.6. UV Absorption

UV absorption ability of dandelion extracts in medium (300 *μ*g/mL) under 280, 290, 300, and 310 nm was measured with a UV-visible spectrophotometer [23].

### 2.7. Cell Staining for MMPs

After treatments, HDFs cultured in 35 mm plates were incubated with 1 *μ*g/mL DQ gelatin (Invitrogen) for 30 minutes to analyze MMP activity under a fluorescent microscope. ImageJ software was used to quantify fluorescent intensity. DQ gelatin is a fluorogenic substrate used to detect MMP activity [4, 20]. Upon digestion, its green fluorescence is revealed to be used to measure MMP activity.

### 2.8. Determination of mRNA Level

Total RNA of HDFs was isolated using TriReagent (Invitrogen) [24]. First strand cDNA was prepared by reverse transcription using M-MuLV reverse transcriptase and random hexamer primers according to manufacturer's protocol (New England Biolabs, Pickering, ON). Real-time PCR was performed in an iCycler iQ^5^ apparatus (Bio-Rad, Mississauga, ON) associated with the iCycler optical system software (version 3.1) using SYBR Green PCR Master Mix, as described previously. The primers of glutathione reductase (GR) were 5′-AGGGCCGCCGTGGTGGAGAGC-3′ (forward, position 265–285) and 5′-ATGGGACTTGGTGAGATTGTTTTG-3′ (reverse, position 475–498). These primers produced a product of 234 bp. The primers of *β*-actin were purchased from Ambion (Streetsville, ON), which produce a product of 295 bp. A standard curve was constructed using a series of dilution of total RNA (Ambion) transcribed to cDNA using the same protocol outlined above to confirm the same amplifying efficiency in the PCR. A standard melting curve analysis was performed using the following thermal cycling profile: 95°C for 10 s, 55°C for 15 s, and ramping to 95°C at 1° increments to confirm the absence of primer dimers. Relative mRNA quantification was calculated by using the arithmetic formula 2^−ΔΔCT^, where ΔCT is the difference between the threshold cycle of a given target cDNA and an endogenous reference *β*-actin cDNA.

### 2.9. Cell Ageing

Cell ageing was evaluated with senescence-associated *β*-galactosidase (SA-*β*-gal) staining [18, 24]. HDFs cultured in 35 mm plates were treated with H_2_O_2_ (100 *μ*M) with or without dandelion extracts (300 *μ*g/mL) for 72 hours. After that, the cells were fixed and stained with X-gal at pH 6.0 overnight. Nuclei were stained with 4′,6-diamidino-2-phenylindole (DAPI, 1 *μ*g/mL) for cell counting. The percentage of ageing cells was calculated as the ratio of blue-stained cells to total cells counted. More than 600 cells were counted from each group.

### 2.10. Materials and Data Analysis

All chemicals were purchased from Sigma (St. Louis, MO) unless stated otherwise. Student's *t*-test in Microsoft excel was used to analyze data between control and treatment group, and the data were presented as mean ± standard error of the mean. Experiments were repeated a minimum of 3 times. Statistical significance was set at *P* < 0.05.

## 3. Results

### 3.1. Dandelion Leaf and Extracts Protect UVB Irradiation-Induced Cell Death

**Figure 1:**
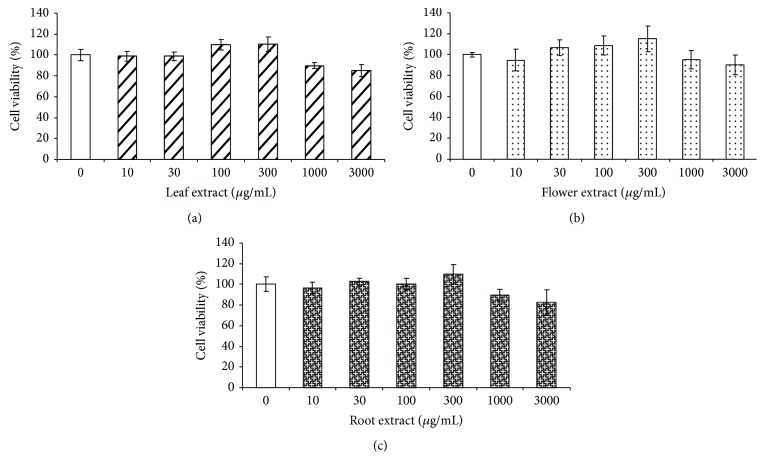
Dandelion extracts have no effect on HDFs cell viability. HDFs were treated with the extracts of dandelion leaf (a), flower (b), and root (c) at 10–3000 *μ*g/mL for 24 hours, and cell viability was then measured with MTT method. *n* = 4.

**Figure 2:**
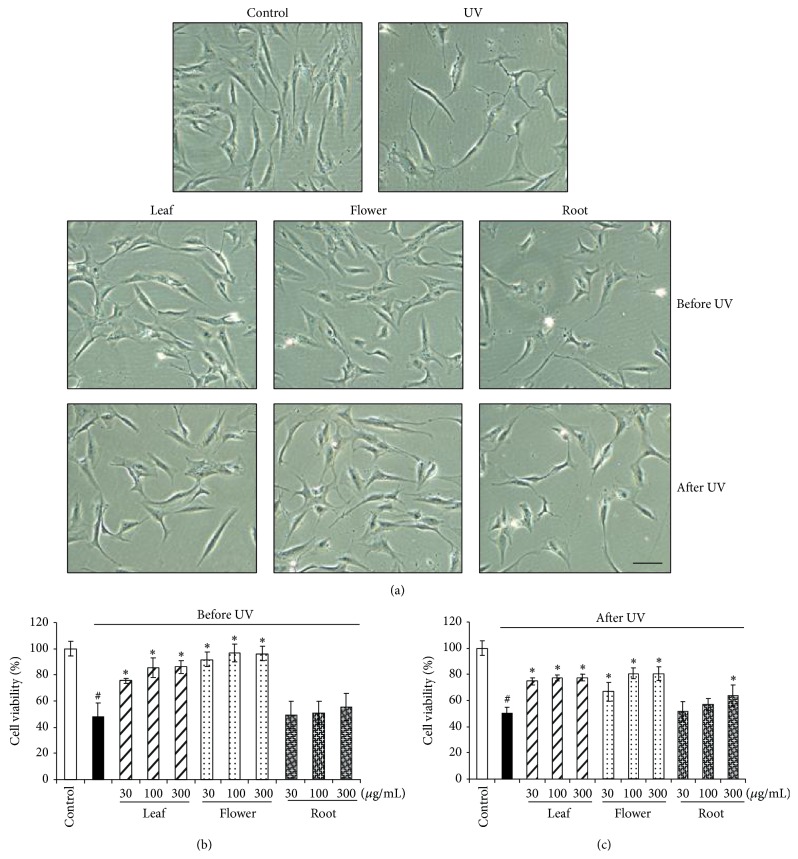
Dandelion leaf and flower extracts protect HDFs from UVB irradiation-induced damage. Dandelion extracts at the indicated concentrations were added 30 minutes before UVB (200 mJ/cm^2^) (b) or promptly after UVB radiation (c). Cell viability was measured by MTT 24 hours after culture. ^#^ *P* < 0.05 versus control; ^*^ *P* < 0.05 versus UVB only. Images in (a) were taken under microscope showing UVB irradiation damages cells, but dandelion leaf and flower extracts (300 *μ*g/mL) protected the cells added from either before or after UVB radiation. Under UVB irradiation, the cells became smaller and less in number. Scale bar: 20 *μ*m. *n* = 3.

The dandelion extracts were prepared from dandelion leaves, flowers, and root, separately, and the extract rate was 10.6%, 11.2%, and 12.7%, respectively. Dandelion extracts alone at 10–3000 *μ*g/mL had no effect on HDFs cell viability (Figure 1). When dandelion extracts (30, 100, and 300 *μ*g/mL) were added into HDFs for 30 minutes before UVB irradiation (Figures 2(a) and 2(b)) or when added promptly after irradiation (Figures 2(a) and 2(c)), leaf and flower extracts significantly protected UVB irradiation-inhibited cell viability. Dandelion root extracts had less effect on UVB-induced cell death when added into HDFs 30 minutes before UVB irradiation. Although supplement of dandelion root extract at 300 *μ*g/mL immediately after irradiation significantly protected HDFs from UVB-induced cell damage, it was quite inefficient compared with the same dose of leaf and flower extracts (Figure 2(b)).

### 3.2. Dandelion Leaf and Flower Extracts Inhibit UVB Irradiation-Stimulated MMP Activity and Oxidative Stress

**Figure 3:**
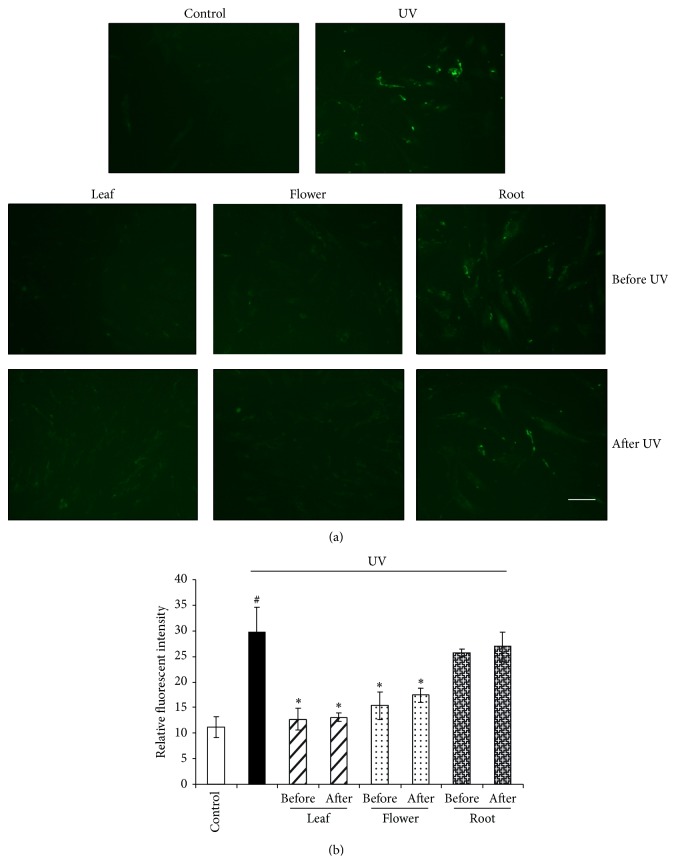
Dandelion leaf and flower extracts inhibit UVB irradiation-stimulated MMP activity. UVB irradiation strengthened the fluorescent intensity of DQ gelatin, reflecting higher MMP activity; however, dandelion leaf and flower extracts (300 *μ*g/mL) significantly decreased MMP activity when added both before and after UVB irradiation. Scale bar: 20 *μ*m. (b) was the statistical analysis from (a) by imageJ software. ^#^ *P* < 0.05 versus control; ^*^ *P* < 0.05 versus UVB only. *n* = 3. Scale bar: 20 *μ*m.

**Figure 4:**
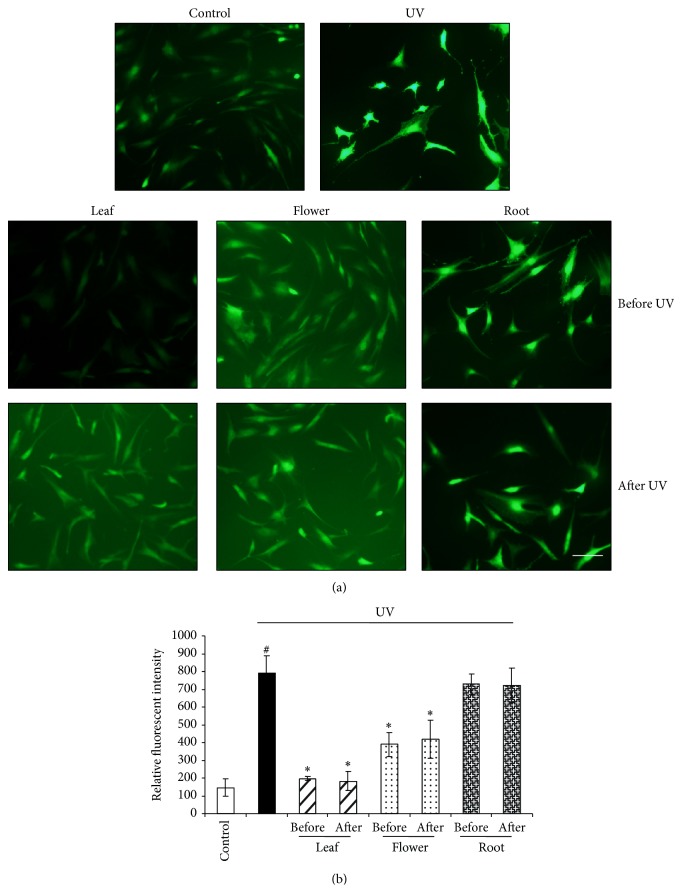
Dandelion leaf and flower extracts inhibit UVB irradiation-induced ROS production. UVB irradiation caused significant increases in the fluorescent intensity of H_2_-DCFDA, reflecting the higher level of ROS; however, dandelion leaf and flower extracts (300 *μ*g/mL) significantly decreased UVB irradiation-induced ROS generation when added both before and after UVB irradiation. (b) was the statistical analysis from (a) by imageJ software. ^#^ *P* < 0.05 versus control; ^*^ *P* < 0.05 versus UVB only. *n* = 3. Scale bar: 20 *μ*m.

UVB irradiation strengthened the fluorescent intensity of DQ gelatin (Figure 3) and H_2_-DCFDA (Figure 4), reflecting the higher activities of MMPs and higher level of ROS, which leads to degradation of ECM and induction of cell death [4, 13]. Dandelion leaf and flower extracts (300 *μ*g/mL) significantly reversed UVB irradiation-induced MMP activity and ROS generation when added either before or after UVB irradiation (Figures 3 and 4). Dandelion root extracts showed less action on protecting HDFs from UVB irradiation-induced MMP activity and ROS generation.

### 3.3. Dandelion Leaf and Flower Extracts Stimulate GSH Generation and GR mRNA Expression

**Figure 5:**
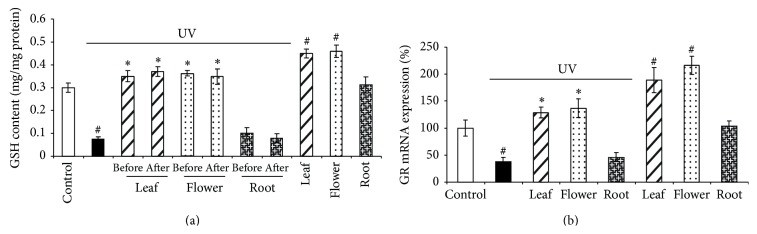
Dandelion leaf and flower extracts stimulate GSH generation and the expression of GR. (a) Dandelion leaf and flower extracts protected UVB irradiation-decreased GSH level. Dandelion extracts (300 *μ*g/mL) were added 30 minutes before UVB or promptly after UVB radiation, and GSH was measured after additional 24-hour culture. ^#^ *P* < 0.05 versus control; ^*^ *P* < 0.05 versus UVB only. *n* = 3. (b) Dandelion leaf and flower extracts stimulated GR mRNA expression. Dandelion extracts (300 *μ*g/mL) were added 30 minutes before UVB, and GR mRNA was measured after additional 24-hour culture with real-time PCR. ^#^ *P* < 0.05 versus control; ^*^ *P* < 0.05 versus UVB only. *n* = 3.

GSH is master antioxidant in our body [24]. We observed that GSH level is significantly lower in UVB-irradiated HDFs (0.075 mg/mg protein), which is completely reversed by dandelion leaf and flower extracts but not dandelion root extracts. Dandelion leaf and flower extracts alone also significantly increased GSH level (0.45 mg/mg protein and 0.46 mg/mg protein, resp.) compared with the control cells (0.3 mg/mg protein) (Figure 5(a)). GR reduces glutathione disulfide to form GSH and is an important GSH-maintaining gene [25]. The mRNA expression of GR was decreased by 62.0% in UVB-irradiated HDFs in comparison with the control cells (Figure 5(b)). Consistent with the GSH data, incubation of HDFs with dandelion leaf and flower extracts significantly induced GR mRNA expression by 29.1% and 37.2% compared with the control HDFs even in the presence of UVB irradiation. In addition, dandelion leaf and flower extracts alone stimulated GR mRNA expression by 89.4% and 116.2%, respectively. Dandelion root extract had no effect on GR mRNA expression in the presence or absence of UVB irradiation (Figure 5(b)).

### 3.4. Dandelion Leaf and Flower Extracts Help UV Absorption

**Figure 6:**
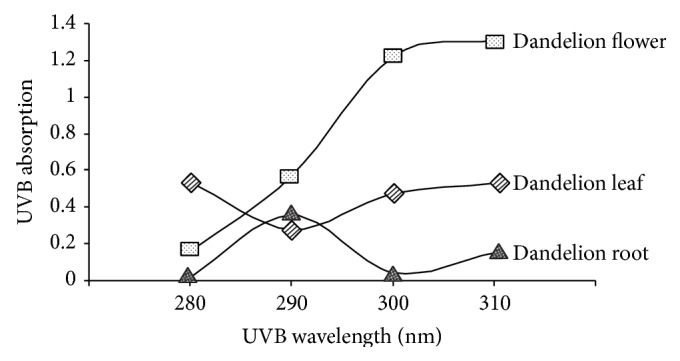
Dandelion extracts help absorb UV irradiation. At wavelengths of 300 nm and 310 nm, flower and leaf extracts had the highest UV absorption capacity. UV absorption of dandelion extracts (300 *μ*g/mL) was measured with a UV spectrometer at 280, 290, 300, and 310 nm, respectively. *n* = 3.

As shown in Figure 6, dandelion extracts could help absorb UV irradiation. At the wavelengths of 300 nm and 310 nm, flower and leaf extracts had the highest UV absorption capacity. At 290 nm, dandelion root extracts displayed higher UV absorption ability compared with dandelion leaf extract but significantly lower than dandelion flower extract. These data suggest the protective role of dandelion extracts against UV irradiation is partially through UV absorption.

### 3.5. Dandelion Leaf and Flower Extracts Protect H_2_O_2_-Induced Cell Ageing

**Figure 7:**
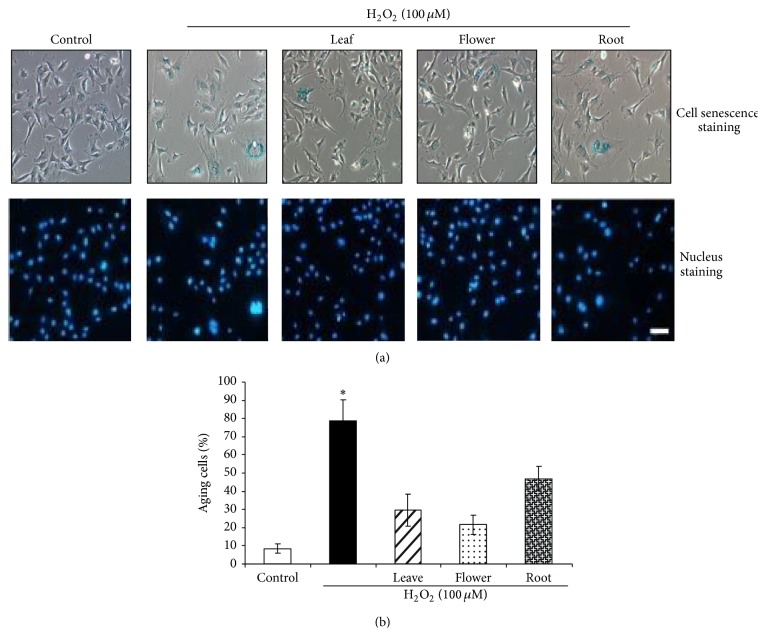
Dandelion leaf and flower extracts protect H_2_O_2_-induced cell ageing. (a) The images showed that H_2_O_2_ provokes cell ageing, which is protected by dandelion extracts. The ageing cells had higher gal activity and stained with blue color. HDFs were also incubated with DAPI for nucleus staining for cell counting. Scale bar: 20 *μ*m. (b) was the statistical analysis from (a). HDFs were treated with H_2_O_2_ (100 *μ*M) with or without dandelion extracts (300 *μ*g/mL) for 72 hours. The percentage of aged cells was calculated from the number of blue-stained cells to total cells counted. ^*^ *P* < 0.05 compared all other groups.

Oxidative stress has been considered one of crucial factors associated with cellular senescence. To study the protective roles of dandelion extracts on oxidative stress-induced premature senescence, we treated HDFs with H_2_O_2_, one main source of oxidative stress. HDFs became enlarged and flattened with a decreased nucleus-to-cytoplasm ratio after H_2_O_2_ (100 *μ*M) treatment for 72 hours (Figure 7(a)), showing the typical characteristics of senescent cells [18, 21]. The cells were then stained for SA-*β*-gal, and the data showed that 78.6% of cells in the H_2_O_2_-treated group become aged (stained with blue); however, dandelion leaf, flower, and root extracts (300 *μ*g/mL) significantly protect H_2_O_2_-induced cell ageing by 61.8%, 73.3%, and 40.0%, respectively (Figure 7(b)).

## 4. Discussion

In recent years, interest on the research of herb medicine has increased all over the world. Many herb extracts have shown therapeutic properties as reported elsewhere [2, 6, 19, 20, 23]. Dandelion is one of the most common and recognizable herbs and is found in almost every part of the world. Many studies have shown that dandelion extracts have a wide range of pharmacological activities, including anticarcinogenic, antioxidant, anti-inflammatory, and antiheart burn activities [10, 11, 16]; however, there are limited scientific studies investigating the antiageing and anti-UV activity of dandelion extracts and very little is known about the mechanisms of action.

Skin ageing is mainly attributed to extrinsic (photoageing) and intrinsic (chronological ageing) processes that are often manifested by increased wrinkles, loss of tensions and elasticity, and altered pigmentation, and so forth [1, 8]. Dermal fibroblasts are within the dermis layer of skin and are responsible for generating connective tissues, including laminin and fibronectin which comprise the ECM [5]. In this study, we found that exposure of HDFs to UVB leads to reduced cell viability. Without dermal fibroblasts, the skin cannot properly recover from injury. Recognition of supplementing natural antioxidant components in skin care is growing important among dermatologists and other medical professionals. Sunscreens are considered to be the gold standard for protecting skin from UV damage. It is recently noted that sunscreen ingredients may become free radicals themselves when activated by UV radiation. In addition, sunscreen chemicals may be absorbed into the skin, causing harmful effect [9]. Here we demonstrated that supplement of dandelion leaf and flower extracts at as lower as 30 *μ*g/mL rescued the cells from UVB-induced cell death, suggesting dandelion may be useful for preventing and treating skin photoageing. More importantly, dandelion extracts produce protective action when added at both before and after UVB irradiation, pointing to the convenient use of this new product. The dandelion extracts at the concentration as higher as 3 mg/mL did not affect cell growth, so dandelion leaf and flower water extracts would be a green choice for development of novel sunscreens.

It is well established that cutaneous exposure to UV irradiation causes higher activities of MMPs and excessive production of ROS [4, 8, 26]. Our studies further validated that UVB irradiation in HDFs induces significantly higher MMP activities and ROS level. Increased activation of MMPs causes the degradation or disorganization of skin connective tissues, which would lead to increased skin wrinkles and loss of skin tone [5]. Oxidative stress is one of the primary causes of skin ageing, and it is believed that oxidative stress is caused by an imbalance between the production of ROS and skin cell's ability to clean up the reactive intermediates [24]. This is really the case, and the present study found that UVB irradiation markedly decreased the content of GSH, a master antioxidant inside the cells. The reduced expression of GR, a GSH producing gene, would be attributed to the lower level of GSH by UVB irradiation [25]. With less GSH generation, the cells will have less ability to remove overproduced ROS. Excitedly, dandelion leaf and flower extracts attenuated UVB-induced MMP activation and ROS generation, with leaf extracts having higher efficiency. Incubation of HDFs with either dandelion leaf or flower extracts alone significantly increased GR mRNA expression and GSH generation. Even in the presence of UVB irradiation, dandelion leaf and flower extracts maintained intracellular GSH level unchanged. All these data indicate that dandelion leaf and flower extracts can protect HDFs from UVB-induced cell death by increasing GSH generation, inhibiting MMP activities and ROS generation.

Ageing at the cellular level is known as cellular senescence [18, 24]. Oxidative stress has been considered one of crucial factors associated with cellular senescence. Most of the senescent cells have flattened cell morphology, promiscuous gene expression, a proinflammatory secretory response, and positive SA-*β*-gal staining at pH 6.0 [18]. Exposure of HDFs to lower doses of H_2_O_2_ (100 *μ*M) for 72 hours induced premature senescence, as evidenced by enlarged and flattened cell morphology with a decreased nucleus-to-cytoplasm ratio, and most of the cells were stained blue for SA-*β*-gal, the typical characteristics of senescent cells. All these changes by H_2_O_2_ in HDFs were significantly reversed by dandelion extracts. Dandelion extracts improve cellular ageing possibly by directly altering the expressions of senescence-related genes, such as p53, or indirectly lowering oxidative stress.

Unlike dandelion leaf and flower extracts, dandelion root extracts had less effect on UV protection. Compared to dandelion roots, dandelion leaves and flowers are characterized by higher polyphenol contents [10]. So it is highly possible that polyphenols but not other components contribute to the protective role of dandelion leaf and flower extracts against UV damage. Most of the components of dandelion leaf and flower extract have been isolated and identified, and some of the important components of the extract, including sesquiterpene lactones and phenylpropanoids, are believed to have anti-inflammatory, antioxidative, and anticancer properties. Whether these components are also playing the critical roles in protecting HDFs from UVB damage needs to be tested. Other components which have not been fully characterized also need to be explored.

In conclusion, dandelion water extracts are able to protect HDFs against UVB damage, before irradiation and also when added promptly after irradiation, via increased UV absorption and reduced MMP activity and oxidative stress. The extracts are also able to prevent oxidative stress-induced premature senescence; however, with both UV protection and antiageing, the root extracts have the smallest effect compared with leaf and flower extracts. These findings can lead to more effective, safer, and environmentally friendly antiageing and UV protection. Future studies, both preclinical and clinical, will be necessary to test the efficacy of dandelion extracts for photoageing.

## Abbreviations

ECM: Extracellular matrix
HDF: Human dermal fibroblasts
GR: Glutathione reductase
GSH: Glutathione
MMP: Matrix metalloproteinase
MTT: 3-(4,5-Dimethylthiazol-2-yl)-2,5-diphenyltetrazolium bromide
PBS: Phosphate buffered saline
ROS: Reactive oxygen species
SA-*β*-gal: Senescence-associated *β*-galactosidase
UV: Ultraviolet.
